# *Vibrio* Colonization Is Highly Dynamic in Early Microplastic-Associated Biofilms as Well as on Field-Collected Microplastics

**DOI:** 10.3390/microorganisms9010076

**Published:** 2020-12-30

**Authors:** Katharina Kesy, Matthias Labrenz, Brittan S. Scales, Bernd Kreikemeyer, Sonja Oberbeckmann

**Affiliations:** 1 Biological Oceanography, Leibniz Institute for Baltic Sea Research Warnemuende (IOW), 18119 Rostock, Germany; matthias.labrenz@io-warnemuende.de (M.L.); brittan.scales@io-warnemuende.de (B.S.S.); 2 Institute of Medical Microbiology, Virology and Hygiene, University Medical Center Rostock, 18055 Rostock, Germany; bernd.kreikemeyer@med.uni-rostock.de

**Keywords:** *Vibrio*, biofilms, microplastics, colonization, co-occurrence networks

## Abstract

Microplastics are ubiquitous in aquatic ecosystems and provide a habitat for biofilm-forming bacteria. The genus *Vibrio*, which includes potential pathogens, was detected irregularly on microplastics. Since then, the potential of microplastics to enrich (and serve as a vector for) *Vibrio* has been widely discussed. We investigated *Vibrio* abundance and operational taxonomic unit (OTU) composition on polyethylene and polystyrene within the first 10 h of colonization during an in situ incubation experiment, along with those found on particles collected from the Baltic Sea. We used 16S rRNA gene amplicon sequencing and co-occurrence networks to elaborate the role of *Vibrio* within biofilms. Colonization of plastics with *Vibrio* was detectable after one hour of incubation; however, *Vibrio* numbers and composition were very dynamic, with a more stable population at the site with highest nutrients and lowest salinity. Likewise, *Vibrio* abundances on field-collected particles were variable but correlated with proximity to major cities. *Vibrio* was poorly connected within biofilm networks. Taken together, this indicates that *Vibrio* is an early colonizer of plastics, but that the process is undirected and independent of the specific surface. Still, higher nutrients could enhance a faster establishment of *Vibrio* populations. These parameters should be considered when planning studies investigating *Vibrio* on microplastics.

## 1. Introduction

*Vibrio* bacteria are omnipresent in the marine ecosystem and often associated with other organisms, such as algae or zooplankton [1]. Some marine *Vibrio* species have the potential to be pathogenic in a human host, such as *V. parahaemolyticus*, *V. vulnificus*, and *V. alginolyticus*. *Vibrio* populations in general, and potentially pathogenic *Vibrio* in particular, are strongly affected by environmental conditions, mostly temperature and salinity [2,3,4].

When microplastics in the ocean became an emerging research topic, a scientific discussion began on the role of microplastics as abiotic carriers for *Vibrio* sp. Following a report of a polypropylene particle from the North Atlantic colonized with a *Vibrio* strain in high abundance [5], several studies addressed this issue. Across the world, potentially pathogenic *Vibrio* species were isolated from plastic debris [6,7,8] or detected by specific PCR [9]. Overall, *Vibrio* abundances on plastic debris appeared low [10,11], especially when compared to natural particles [12]. Recent studies, however, point out the necessity to include abiotic and biotic factors in *Vibrio*-related investigations. For instance, Foulon and colleagues [13] showed in colonization experiments with *V. crassostreae* a long attachment (6 days) on irregular polystyrene beads as compared to smooth particles (<10 h). In 2019, both Kesy et al. [14] and Li et al. postulated a positive correlation of salinity and *Vibrio* numbers on plastic. In high-salinity waters ≥ 26 PSU *Vibrio* abundance was 2–10 times higher than in seawater or in sediment, while no difference was detected in less saline waters [15].

While many open questions remain, it is clear that microplastics and *Vibrio* bacteria share a habitat. Our study area, the Baltic Sea, is a brackish, enclosed-shelf sea system in Northern Europe with a salinity gradient of appr. 25 to 3 PSU. The system is under severe anthropogenic pressure with regard to pollution as well as the consequences of climate change. Microplastics reach the Baltic Sea due to irresponsible handling of plastic litter and mismanaged municipal wastewater [16,17], and potentially pathogenic *Vibrio* species are increasing in abundance due to warmer water temperatures, which in turn increases the infection risk [18]. The interactions between microplastics and *Vibrio* sp. in the Baltic Sea, however, are not fully understood.

Here, we report *Vibrio* abundances from an in situ experiment focusing on early colonization, as well as field sampled microplastics from the Baltic Sea. We set out to elucidate the role, dynamics, and influence of biotic and abiotic factors on the potential of *Vibrio* colonization on microplastics to promote the discussion and provide future directions in the study of *Vibrio* bacteria and microplastics.

## 2. Material and Methods

### 2.1. In Situ Incubation Experiment

**Figure 1:**
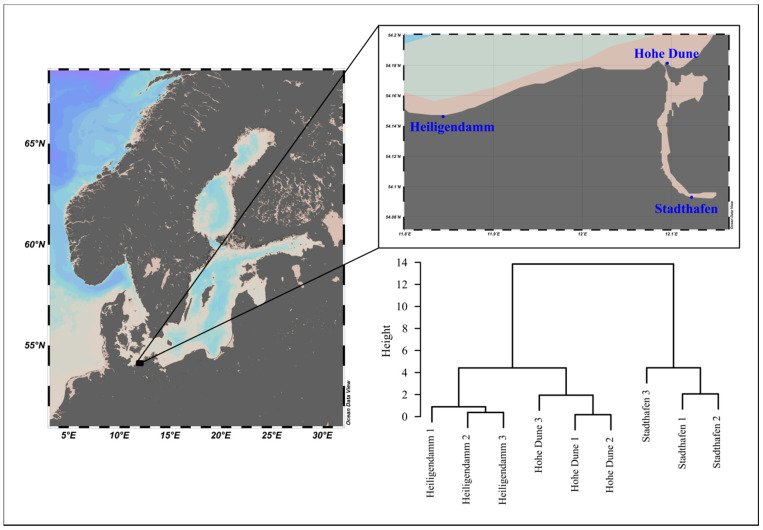
Sampling sites of the in situ incubation experiment, showing the Baltic Sea in Northern Europe (**left**) and exact location along the coast and Warnow river (**right**). The dendrogram depicts hierarchical clustering of sites based on Euclidian distance of z-transformed physicochemical and nutrient data. Clusters were agglomerated using Ward’s method. Maps were constructed using Ocean Data View v. 5.0.0 [24].

Two different plastic resin pellets (ø 3 mm), polyethylene (PE, HDPE HTA108, density 0.961 g·cm^−3^, ExxonMobil, Irving, TX, USA) and polystyrene (PS, 143 E, density 1.04 g·cm^−3^, BASF, Ludwigshafen, Germany), were incubated at 3 sites, 1 in the Warnow river at the recreational harbor of the city of Rostock, Germany (Stadthafen), and 2 coastal station of the Baltic Sea in May 2019. One station was close to the Warnow estuary and within the recreational harbor of Hohe Dune, and one at the long-term observatory site of the pier of Heiligendamm (Figure 1). Thirty grams of PE pellets and 35 g of PS pellets were incubated in triplicates within the first meter of the water column, using in-house developed incubators as described in Oberbeckmann et al. (2018) [19]. A subsample of pellets (~7.5 g for PE, ~8.5 g for PS) was taken after 1, 5, and 10 h of exposure. For that, the incubators were retrieved and stored in a bucket with local water while being opened and the pellets collected using a sterilized spoon. The pellets were collected in 50 mL Falcon tubes filled with sterile NaCl solution (2, 10, and 14 g/kg, respectively), and gently inverted several times to remove loose cells. The NaCl solution was discarded carefully and the procedure repeated 2x. After that, the samples were shock-frozen in liquid nitrogen and stored at −80 °C until DNA extraction.

Three water samples (500 mL) were taken during the course of the experiment and serially filtered on site through a 3 µm (seston-attached community) and a 0.22 µm cellulose nitrate filter (GE Whatman, Little Chalfont, UK) (free-living community). Filters were shock-frozen and stored as described above.

Additionally, water samples (1 L) were taken for chlorophyll a, nutrient, and organic material content and treated as described below.

### 2.2. Field-Collected Plastic Particles

Microplastics were collected during a Baltic Sea cruise (POS488, August–September 2015) from surface waters by Manta net trawl with a 300 µm mesh size. The mesh’s cod-end was then rinsed with sterile-filtered seawater, and individual particles were picked after visual inspection with sterile tweezers, transferred into a sterile microcentrifuge tube, shock-frozen in liquid nitrogen, and stored at −80 °C until further processing. One liter to 500 mL of surface water from each station was also collected and serially filtered as described for the in situ incubation. Nutrients were measured on board with standard colorimetric methods [20], and samples for chlorophyll a concentrations, organic content, and nutrient content were filtered on board, stored frozen at −80 °C and −20 °C, respectively and analyzed at the Institute’s laboratory following standard procedure [20,21,22]. After DNA extraction, particles were confirmed to be of plastic polymer origin using attenuated total reflection (ATR) Fourier transform infrared (FTIR) spectroscopy as described in Lorenz et al. (2019) [23].

### 2.3. DNA Extraction, Library Preparation and Sequencing

DNA was extracted from 40 pooled in situ incubated PE or PS particles, respectively. For the field-collected particles, DNA was extracted from each individual particle. Phenol-chloroform extraction followed a modified protocol from Anderson and MacKay (1983) [25] as described in Oberbeckmann et al. (2018) [19]. Empty tubes were included from the beginning of the extraction including sequencing as blank extraction controls. In a previous study, it was shown that sequences retrieved from the raw test materials were negligible; thus, raw material samples were not included in this experiment [14].

DNA concentrations were adjusted to the average lowest DNA in the dataset. The V3 and V4 variable region of the 16s rRNA gene was amplified using modified primers from Takahashi et al. (2014) [26]: Pro341-XT: 5’-TCGTCGGCAGCGTCAGATGTGCAGCCTACGGGNBGCASCAG-3’ and Pro805-XT: 5’-GTCTCGTGGGCTCGGAGATGTCTACNVGGGTATCTAATCC-3’ and the Kapa HiFi HotStart ReadyMix. PCR conditions were as follows: 3 min of denaturation at 95 °C, followed by 25 cycles of 95 °C for 30 s, 55 °C for 30 s, 72 °C for 30 s, and a final elongation at 72 °C for 5 min. Positive PCR products were confirmed by agarose gelelectrophoresis (1.2% *w/v* in 1x TBE buffer) and stored at −20 °C until further processing. From here, the Illumina protocol ”16S Metagenomic Sequencing Library Preparation” was followed, and libraries were sequenced on an Illumina MiSeq using a 600 cycle V3 chemistry kit. Positive and negative controls were included in each run.

### 2.4. Sequence Processing and Downstream Data Analysis

The sequences were processed using the mothur pipeline v. 1.39.5 and v. 1.40.0 [27] following the mothur standard operating procedure (SOP) [28,29] with subsequent modifications, and the sequences were quality filtered using the following parameters (in situ incubation/field-collected dataset): permitted sequence length = 400–430/420–480 bp; maximum number of ambiguous bases per sequence = 0; maximum number of homopolymers per sequence = 8. The sequences were dereplicated while allowing for a mismatch of 4 nucleotides and taxonomically assigned with Wang classification and the SILVA SSU Ref release 132/123 database [30] (required bootstrap value ≥ 85/80%).

For both datasets, sequences classified as *Eukaryota*, Mitochondria, *Archaea*, chloroplasts, or unclassified were removed. Finally, operational taxonomic units (OTUs) were picked at a 97% sequences similarity threshold. The representative sequences for each OTU were retrieved using the get.oturep command with method = “abundance”. Raw sequences obtained in this study were submitted to the NCBI Sequence Read Archive (SRA) under BioProjects PRJNA678316 (in situ incubation experiment) and PRJNA632000 (Baltic Sea cruise data).

The resulting OUT table, taxonomy table, representative OUT sequences, and metadata were all imported into the R environment for statistical computing v. 3.5.1 [31]. Further data analysis was conducted using the packages “phyloseq” v. 1.26.1, “speedyseq” v. 0.1.2.9004, “decontam” v. 1.2.1, “reshape2”, “ggplot2” v. 3.3.0, and “vegan” v. 2.5-6 [32,33,34,35,36,37]. For the in situ incubation data, OTUs with ≤ 3 reads, as well as those OTUs with > 3 reads in the positive control, were discarded from the dataset. Potential contaminant sequences were identified using the blank extractions and negatives as controls in the “isContaminant” function from the “decontam” package (method = “either” and threshold = 0.2) and also discarded. After that, all controls were removed from the dataset.

For the Baltic Sea cruise data, OTUs with a total abundance of ≤ 2 and samples with less than 10,000 reads were removed, during which all laboratory controls were removed from the analysis but one.

Phylogenetic trees (based on neighbor joining with bootstrapping, maximum likelihood, maximum parsimony, and a consensus tree from the 3 methods) with all representative sequences of the family *Vibrionaceae* from both datasets were constructed in ARB v. 6.0.2 [38] and the SILVA SSU Ref release 132, as described in the supplement of Kesy et al. 2019 [14]. Those OTUs that had prior been assigned as unclassified *Vibrionaceae*, but clustered within the *Vibrio* clades in a majority of the calculated trees were also regarded as *Vibrio* OTUs in the subsequent analysis (Figure S1). Since in this study we were only interested in the *Vibrio* community, the 2 datasets were then trimmed to contain only OTUs classified as *Vibrio* spp., either by taxonomic assignment or by their clustering within the calculated trees.

Significant differences between relative abundances of *Vibrio* spp. between timepoints and sites in the in situ incubations were calculated on aggregated *Vibrio* abundances using the Kruskal–Wallis rank sum test and post hoc Conover–Iman test for multiple comparisons, with Benjamini–Hochberg correction applied, with the conover.test package [39]. To measure the variation of *Vibrio* OTU composition among biofilms on PE and PS between sites, we calculated the Bray–Curtis dissimilarities between samples, based on square-root-transformed relative abundance of the *Vibrio* OTUs of each sample. To include the information of replicates with no *Vibrio* OTU, we added a dummy OTU with an abundance of 0.000000001 to all samples. We then used a PCoA to ordinate the samples based on their Bray–Curtis dissimilarities in two-dimensional space, and to calculate the spread of the samples around the group’s spatial median for the factor “site” within the ordination space as a measure of community variability. This was carried out with the PERMDISP routine [40,41] as implemented in vegan’s “betadisper” function. Significant differences in the dispersions around the group’s spatial median between pairs of sites were calculated with the Tukey’s HSD test.

For the Baltic Sea cruise dataset, the shortest distance (km) to a major city (> 100,000 inhabitants, [42]) was calculated with the Haversine formula, using the online calculation tool of the latlongdata.com webpage (https://latlongdata.com/lat-long-converter/, Lat Lng Data, Ontario, Canada, accessed: 22 September 2020). Spearman rank correlations (rho) were calculated on aggregated *Vibrio* abundances, and the shortest distance to a major city, number of particles, physicochemical water parameters, as well as nutrient concentrations.

Co-occurrence networks were constructed for each timepoint from the in situ incubations, including both PE and PS samples as well as from all plastic samples collected in the Gulf of Finland. For all networks, only OTUs that were present with a relative abundance > 0.01% in at least three samples were used. Networks were constructed using the CoNet application within the Cytoscape program [43], as described in Faust and Raes (2016) [44], including environmental parameters. Because some were strongly correlated, only the following parameters were included in the networks: salinity, temperature, PO_4_^3−^, chlorophyll a, dissolved organic carbon (DOC), and dissolved nitrogen (DN). Resulting networks were visualized using the yFiles Organic layout within Cytoscape and analyzed with the NetworkAnalyzer tool.

## 3. Results and Discussion

### 3.1. Vibrio Abundances and Composition Are Highly Variable during the First Hours of Colonization

**Figure 2:**
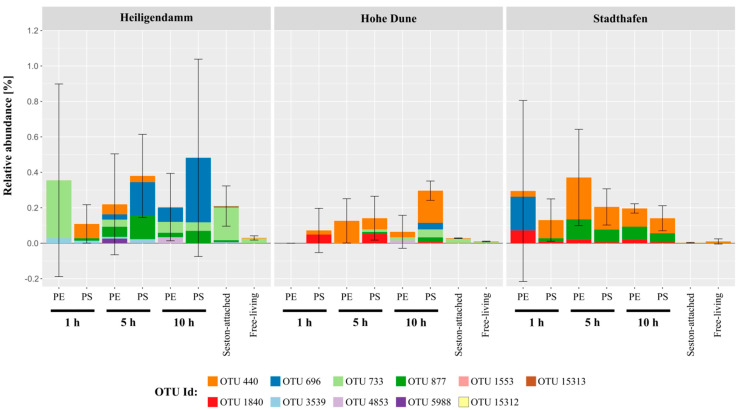
Mean relative abundance data of *Vibrio* operational taxonomic units (OTUs) on PE, PS, and water during an in situ incubation experiment covering the Warnow river (Stadthafen) and two Baltic Sea coastal station (Heiligendamm and Hohe Dune). Plastic samples were taken after 1, 5, and 10 h. *Vibrio* abundances within the water were fractionated into those on the seston-attached fraction (>3 µm) and in the free-living fraction (3–0.22 µm). Colors indicate different *Vibrio* OTUs based on 97% sequence similarity. Error bars give the standard deviation (SD) of the mean aggregated *Vibrio* abundances.

**Figure 3:**
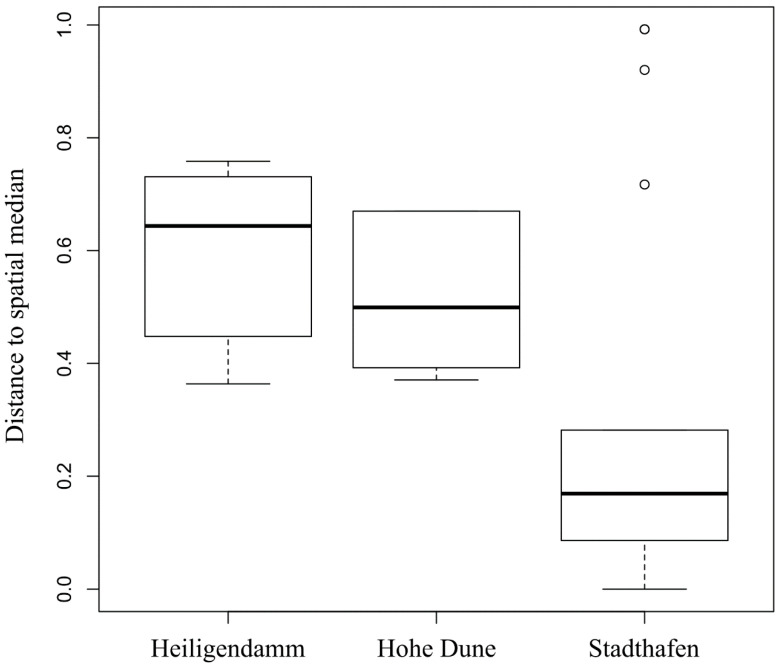
Boxplot depicting the distances to the groups’ spatial medians for the factor “site” of biofilms on PE and PS during the in situ incubation experiment, covering the Warnow river (Stadthafen) and two Baltic Sea coastal stations (Heiligendamm and Hohe Dune), after 1, 5, and 10 h. Groups’ spatial medians were derived from a PCoA-ordination based on Bray–Curtis dissimilarities between *Vibrio* communities as a measure for community variability.

Based on our in situ experiment, colonization of plastics by *Vibrio* commenced within the very first hour of exposure, showing that *Vibrio* bacteria are amongst the very first colonizers on plastics. *Vibrio* colonization was highly dynamic, since there was a high variability in the abundance, as well as the composition of *Vibrio* OTUs within the first hours (Figure 2, Figure 3 and Figure S2). This hints at a rather undirected colonization process, with stochastic events overwhelmingly contributing to the colonization of plastics by *Vibrio*, e.g., the encounter of the material by a *Vibrio* cell.

We observed a trend of higher *Vibrio* abundances on plastics than on the seston-attached or in the free-living community, but due to the great variation even between biological replicates (apparent from the high SD in Figure 2), these differences were overwhelmingly not significant.

Consequent significant differences between both PE and PS, and the water samples, were only found for the inner river station “Stadthafen” after 5 and 10 h (*p*-value between 0.01 and 0.02). When comparing the three locations of the incubation experiment, *Vibrio* composition and abundance became more stable after 1 h at the inner river site “Stadthafen”. While the sites “Stadthafen” and “Hohe Dune” were dominated by OTU 440 and OTU 877, “Heiligendamm” was dominated by OTU 696 and OTU 733 (Figure 2 and Figure S2). These most abundant OTUs clustered closest to *Vibrio* type strains belonging to the *V. anguillarum* clade (OTU 440 and OTU 877), the *V. vulnificus* clade (OTU 696), and the *V. rumoiensis* clade (OTU 733) [45] in the phylogenetic consensus tree (Figure S1). Notably, the first two clades encompass species with pathogenic potential in animal and human hosts [46]. However, species assignment within the genus *Vibrio* based solely on a fragment of the 16S rRNA gene should be treated cautiously, since this marker does not provide sufficient phylogenetic resolution for reliable species identification [47]. Furthermore, it does not allow any prediction about the real pathogenic potential, since this often depends on virulence factors that may or may not be present even within the same species [48,49].

We used the distance to the group’s spatial median for the factor “site” within a PCoA-ordination as a measure for the variability of *Vibrio* OTU composition among plastic biofilms and found that the variability was significantly lower at the inner river site “Stadthafen” compared to ”Heiligendamm” and “Hohe Dune” (*p* = 0.001 and 0.01, Figure 3 and Figure S3).

One factor that could contribute to the variability within the very first hours of colonization could be the hydrodynamic regime [50]. On the sampling day of the coastal station “Heiligendamm”, it was windy, which created much greater turbulences than during sampling at the other two sites. However, during sampling at the site “Hohe Dune”, conditions were extremely calm, but still *Vibrio* communities on plastics at both “Heiligendamm” and “Hohe Dune” were significantly more variable than at the inner city “Stadthafen”. Thus, it is unlikely that water turbulences were a major factor. The “Stadthafen” was the site with highest nutrient concentrations and lowest salinities (Figure 1, Table S1). While salinity is the major factor structuring the occurrence of different *Vibrio* bacteria [2,3,4], higher nutrients could lead to a more rapid succession of the *Vibrio* community, as has already been shown for biofilm communities in the Baltic Sea [19]. Additionally, *Vibrio* cell numbers might generally be higher at sites with higher nutrients and thus there is a greater chance of a cell surface encounter and subsequent establishment of a *Vibrio* biofilm population. A recent study by Sun et al. (2020), investigating biofilms on different plastic material, reported extremely high *Vibrio* abundances on microplastics incubated among a mussel farm, with a mean of ~52% in biofilms and a mean of ~17% in the corresponding water samples. The authors attributed this to the “high temperature and concentration of organic matter released by cultured shellfishes” [51]. This underlines our assumption that higher nutrients could lead to a more rapid establishment of, and perhaps more consolidated, *Vibrio* population on microplastics.

### 3.2. Vibrio Abundances on Field-Collected Particles Correspond with Proximity to Major Cities

**Figure 4:**
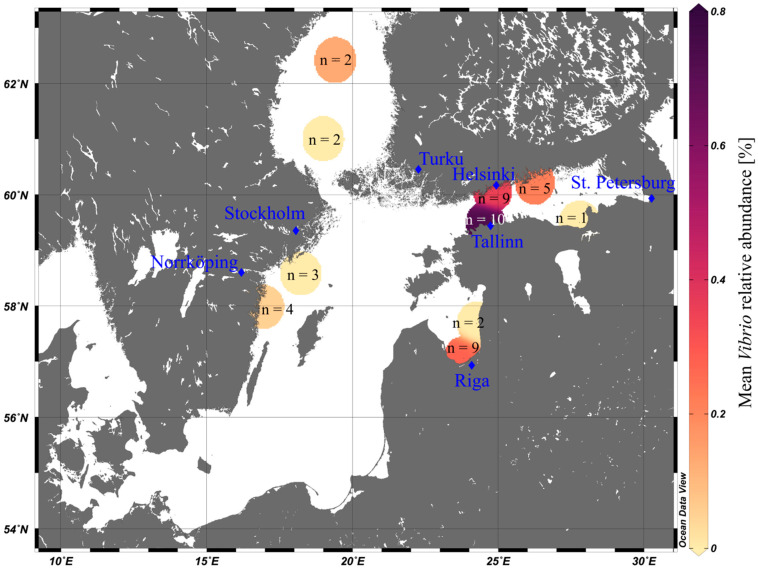
Mean relative abundance data of *Vibrio* spp. on field sampled plastics of the POS488 summer cruise (2015), covering the Gotland Sea (ex. Bay of Gdansk), Gulf of Riga, Gulf of Finland, and Bothnian Sea. Color gradient depicts mean *Vibrio* abundance (%) (in the case of n = 1, no mean was calculated), n = number of microplastic particles. Blue diamonds show major cities (>100,000 inhabitants) around the sampling area. The map was constructed using Ocean Data View v. 5.0.0 [24].

We also analyzed *Vibrio* concentrations on particles sampled from the Baltic Sea with unknown age. *Vibrio* abundances increased close to larger cities (*Vibrio* abundances and distance to cities with > 100,000 inhabitants: rho = −0.77, Figure 4 and Figure S4). This strengthens our assumption that *Vibrio* bacteria are early colonizers of plastics, since cities provide one major pathway of microplastics entering the Baltic Sea, and particles found close to cities probably entered the system more recently [17].

Maximum mean abundance of *Vibrio* was higher (0.8%, Figure 4) on field-collected particles than on the in situ incubated particles (max. 0.5%, Figure 2). Of note, the two dominant OTUs in this dataset also clustered closest with type strains from the *V. anguillarum* and *V. vulnificus* clades, respectively (Figures S1 and S5a). This is particularly interesting, since both datasets are based on data from different years and seasons but show a consistent colonization based on 16S rRNA gene sequence similarity. If these are indeed pathogenic vibrios cannot be determined here.

However, also in this field-based dataset, *Vibrio* abundances varied among particles collected during the same Manta trawl (e.g., between ~3% and 0% at MP18, the station closest to Tallinn; Figure S5a). Moreover, the number of collected microplastics correlated negatively with distance to main cities (rho = −0.91), fitting well with the hypothesis of coastal cities being a potential microplastic source. Distance to a major city and number of particles were factors that correlated most strongly with *Vibrio* numbers (rho = −0.77 and 0.91) compared to other parameters (rho ≤ 0.57, Figure S4). Thus, another parameter to consider when assessing microplastics as vectors for putative pathogenic *Vibrio* is their potential sources, both of the particles and the bacteria. While in most regions of the world’s oceans we do not expect a relevant colonization of microplastics with potentially pathogenic taxa, the risk might be higher in strongly anthropologically impacted areas. A great amount of plastic waste worldwide enters the oceans at coastal sites with high populations, often situated at estuaries with dynamic conditions, and close to waste water drainage systems, such that an enhanced dissemination of potentially pathogenic bacteria on plastics poses a realistic threat to these communities [52].

### 3.3. Vibrio Bacteria Are not Well Connected in Biofilm Networks

**Figure 5:**
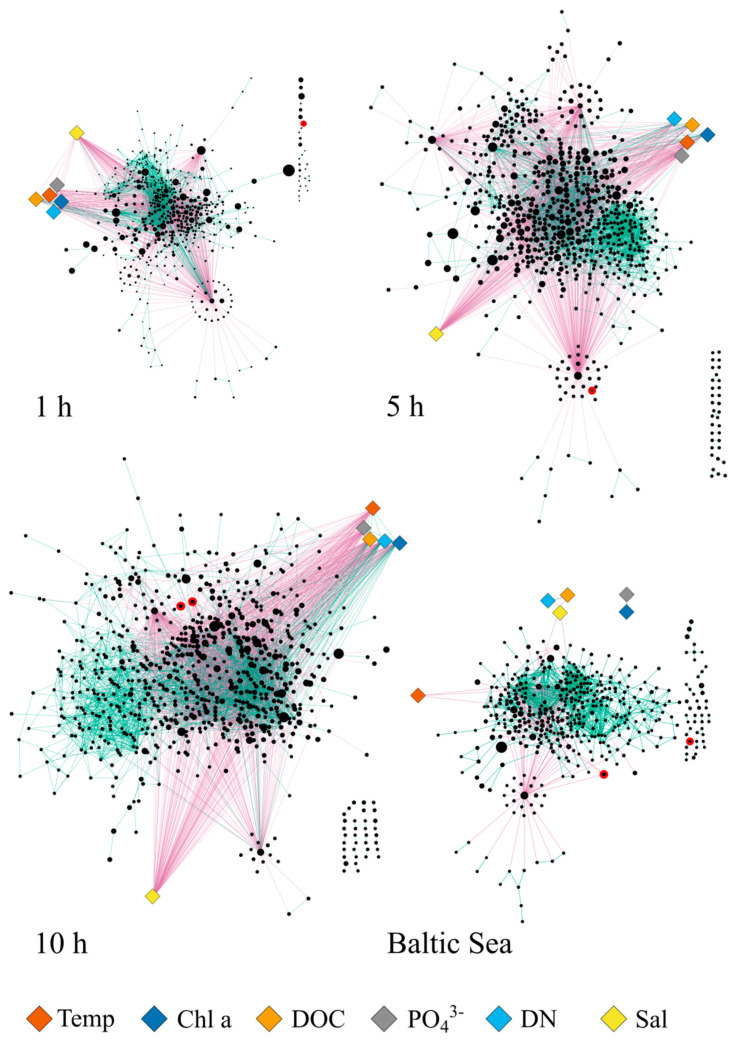
Co-occurrence networks of bacterial OTUs (circles), with relative abundance > 0.01% in at least three samples on plastics. Circle size represents mean abundance across all samples. Co-presence between OTUs is depicted with green edges, mutual exclusions with magenta edges. Co-occurrence networks for the exposure experiment include all stations but separated for the three different timepoints (1, 5, and 10 h), including environmental parameters (diamond shape: Temp = temperature, Chl a = chlorophyll a, DOC = dissolved organic carbon, PO_4_^3−^ = phosphate, DN = dissolved nitrogen, Sal = salinity). A co-occurrence network for field-collected microplastics was constructed for all stations within the Gulf of Finland. *Vibrio* OTUs are highlighted with a red contour.

To gain further insights into the potential co-operations or exclusions of *Vibrio* within the developing biofilm, we constructed co-occurrence networks for each timepoint with OTUs > 0.01% in at least three samples from the in situ experiment. As a comparison, a co-occurrence network from all particles sampled in the Gulf of Finland was also constructed. We found that *Vibrio* bacteria were not well integrated in the networks (Figure 5). While the number of total OTUs within the networks from the in situ experiment increased over time from 510 at 1 h to 686 at 5 h and to 745 after 10 h, only one out of three *Vibrio* OTUs (OTU 1840) was included in the 1 and 5 h networks. In the 10 h network, two Vibrio OTUs out of six that were in the starting dataset were still present in the final network (OTU 440 and OTU 696). Although OTU numbers within the networks increased over time, the average number of neighbors for the complete networks stayed constant across the three timepoints (10 neighbors). The majority of *Vibrio* OTUs had decisively fewer connections to other OTUs than the average, with either only one positive or negative. This was true for all except for OTU 696 in the 10 h network, which had more correlations to other OTUs than the average. However, 24 out of these 25 correlations were negative. The co-occurrence network from the field-collected particles as compared to the in situ experiment had a lower number of total OTUs (431), which may also hint towards an only recent colonization. Here again, we found that from four *Vibrio* OTUs in the dataset for network construction, only two were present in the final network (OTU 406 and OTU 1936), with three negative correlations and one positive correlation, respectively, compared to an average of seven neighbors for the whole network. A similar pattern for *Vibrio* was observed in a study investigating enrichment cultures obtained from floating plastics in the Mediterranean Sea, where *Vibrio* was found to co-occur mostly with a few other *Vibrio* OTUs after 2 months [53]. Feng et al. (2020) [54] reported that on microplastics incubated in coral areas, the genus *Vibrio* was amongst the most abundant OTUs after 2 weeks; however, it was not part of the co-occurrence network constructed from those samples. Thus, *Vibrio*, albeit present in the biofilm, is not well connected within the biofilm network, indicating that this genus is not dependant on resources or secondary metabolites provided by other biofilm bacteria. This also strengthens the assumption that *Vibrio* bacteria are early colonizers of particles. The genus *Vibrio* is known for a “feast or famine” growth strategy [55,56,57], and a new surface may provide a new colonization opportunity which *Vibrio* is quick to respond to [1]. Unfortunately, in this study, the control material (aquarium gravel) did not provide any meaningful data for comparison to other surface types.

However, several studies have shown that *Vibrio* is also abundant in young biofilms (≤7 days) on other substrates, such as chitin, fibreglass, wood, glass, or feathers [8,14,51,58,59].

This again indicates that biofilm formation for *Vibrio* is firstly an undirected process, driven by the opportunity of an available new surface. The absence of *Vibrio* in biofilms from older particles might indicate that *Vibrio* is not long-term attached to plastics, maybe because in later stages of biofilm formation, the potential of the substrate to serve as a nutritional source for *Vibrio* bacteria becomes more important. However, this hypothesis, along with how nutrient concentration may affect long-term proliferation of *Vibrio* on microplastics, would need to be investigated in future studies.

## 4. Conclusions

We agree with Wright et al. (2020) [60] that the analysis of microplastics as a potential vector for putative pathogenic *Vibrio* needs to include the information about their real pathogenic potential. However, our results showed that the colonization of plastics with diverse *Vibrio* can be highly dynamic, especially in lower nutrient and higher saline systems. We therefore propose that future studies investigating *Vibrio* bacteria on (micro-)plastics consider the quality and physicochemical parameters of water and temporal dynamics, as well as proximity to potential sources to be included a priori.

## Data Availability

The data presented in this study are openly available from the NCBI Sequence Read Archive (SRA) under BioProjects PRJNA678316 and PRJNA632000.

## Supplementary Materials

The following are available online at https://www.mdpi.com/2076-2607/9/1/76/s1, Figure S1: Phylogenetic consensus tree of all *Vibrionaceae* OTUs from the in situ incubation experiment and Baltic Sea cruise dataset. Figure S2: Relative *Vibrio* abundance per replicate on PE, PS, and water during the in situ incubation experiment. Figure S3: PCoA-ordination plot based on Bray–Curtis dissimilarities between samples. Figure S4: Spearman rank correlation of mean *Vibrio* abundances on field-collected microplastics, distance to city, number of particles, nutrients, and physicochemical water properties during the Baltic Sea summer cruise 2015. The correlogram was constructed using the corrplot package for R [61] Figure S5: Relative *Vibrio* abundance per replicate of field-collected microplastics and water during the Baltic Sea summer cruise 2015. Table S1: Site descriptions, physicochemical water parameters, and nutrient concentrations during the in situ incubation experiment.
