# Supplementary material for: Vibrio Colonization Is Highly Dynamic in Early Microplastic-Associated Biofilms as Well as on Field-Collected Microplastics
Source: Microorganisms. 2020 Dec 30;9(1):76. doi: 10.3390/microorganisms9010076 (PMC7823642; doi:10.3390/microorganisms9010076)
Supplement: Supplementary file 1 [file microorganisms-09-00076-s001.zip › Supplement_revised.docx]

**Supplementary Material for:**

***Vibrio* colonization is highly dynamic in early microplastic-associated biofilms as well as on field-collected microplastics**

Katharina Kesy^1*^, Matthias Labrenz^1^, Brittan S. Scales^1^, Bernd Kreikemeyer^2^ & Sonja Oberbeckmann^1*^

^1^Biological Oceanography, Leibniz Institute for Baltic Sea Research Warnemuende (IOW), Rostock, Germany

^2^Institute of Medical Microbiology, Virology and Hygiene, University Medical Center Rostock, Rostock, Germany

*Corresponding authors: katharina.kesy@io-warnemuende.de (K.K.)

sonja.oberbeckmann@io-warnemuende.de (S.O.)

**
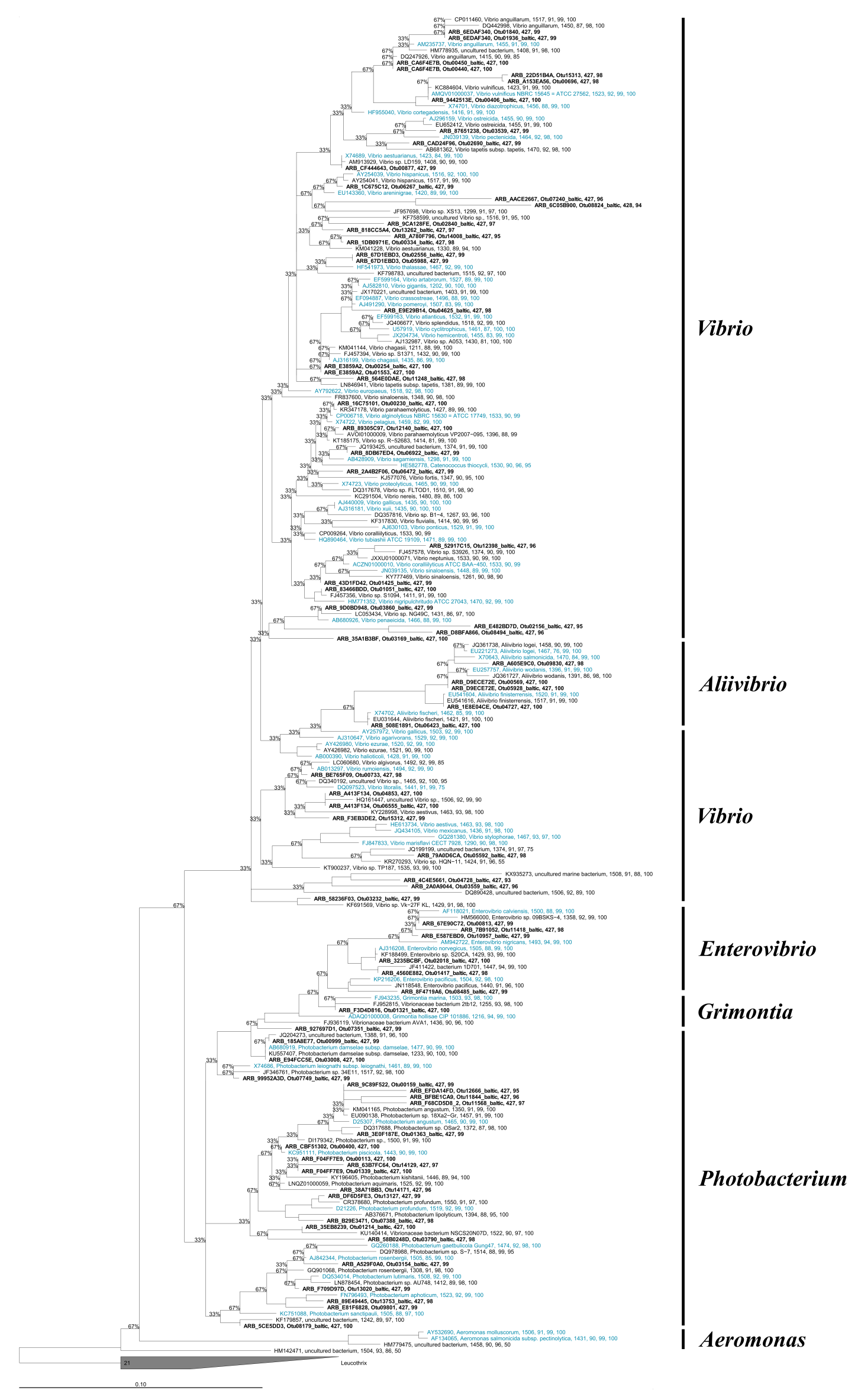
**

Fig. S1 (previous page): Phylogenetic consensus tree of all *Vibrionaceae* OTUs (in bold) from the *in situ* incubation experiment and Baltic Sea (_baltic) cruise dataset, based on a Neighbor joining-, Maximum Likelihood-, and Maximum parsimony-tree. As reference, the Silva SSU Ref Nr. 132 database was used. Blue OTUs depict type strains. Bootstrap values give % in how many trees the branches occurred. The tree was rooted with members of the family *Leucothrix* as outgroup. Bar: 10 substitutions per 100 nucleotides.


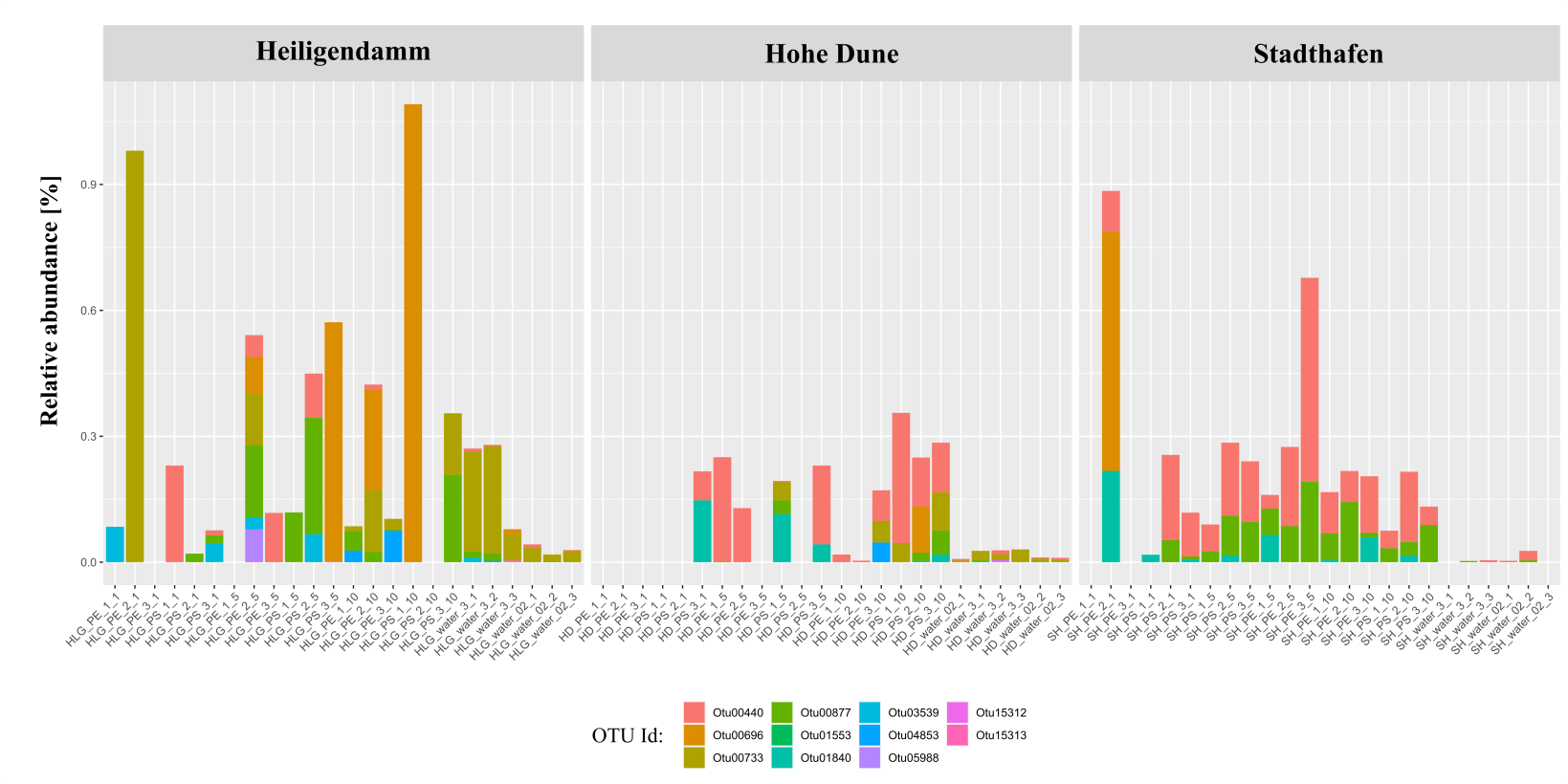


Fig. S2. Relative *Vibrio* abundance (%) per OTU and replicate on PE, PS, and water during an *in situ* incubation experiment covering the Warnow river (Stadthafen) and 2 Baltic Sea coastal stations (Heiligendamm & Hohe Dune). Plastic samples were taken after 1 h, 5 h, and 10 h. *Vibrio* abundances within the water were fractionated into those on the seston-attached fraction (> 3 µm, water_3) and in the free-living fraction (3-0.22 µm, water_02). Colors indicate different *Vibrio* OTUs based on 97% sequence similarity.


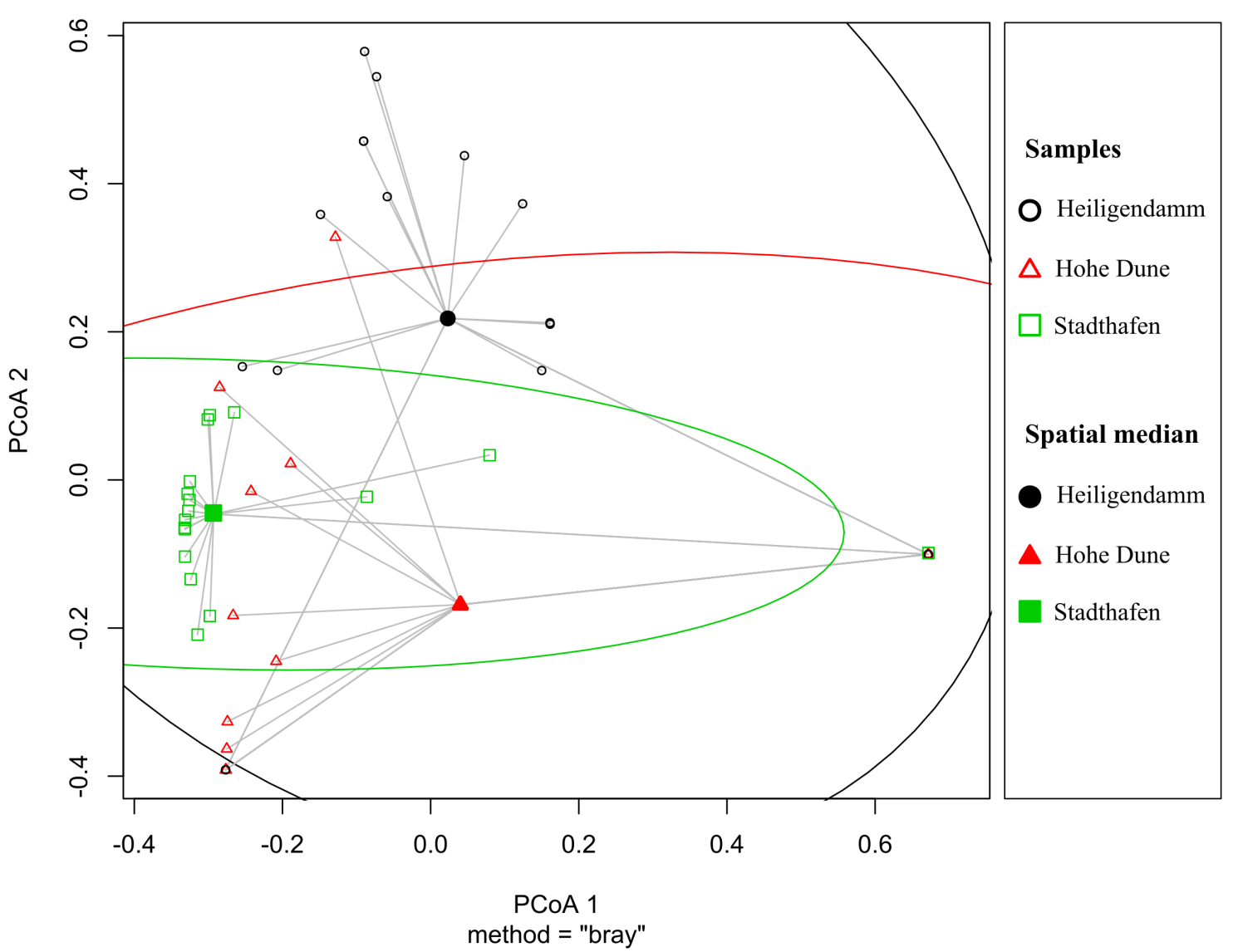


Fig. S3. PCoA-ordination plot based on Bray-Curtis dissimilarities between samples, calculated on square-root transformed relative abundances of the different *Vibrio* OTUs on PE and PS after 1 h, 5 h, and 10 h from the *in situ* incubation experiment. To include samples with no vibrios, a dummy OTU was added to all samples with the same abundance (0.000000001). Symbols and colors depict the different sampling sites: black circles = “Heiligendamm”, red triangles = “Hohe Dune”, green squares = “Stadthafen”. Empty symbols represent the ordination of one sample within the 2-dimensional space; filled symbols represent the spatial median for the grouping factor ‘site’; and grey lines the distance between each sample and its corresponding group median. The ellipses show the 95%-confidence area for each group.


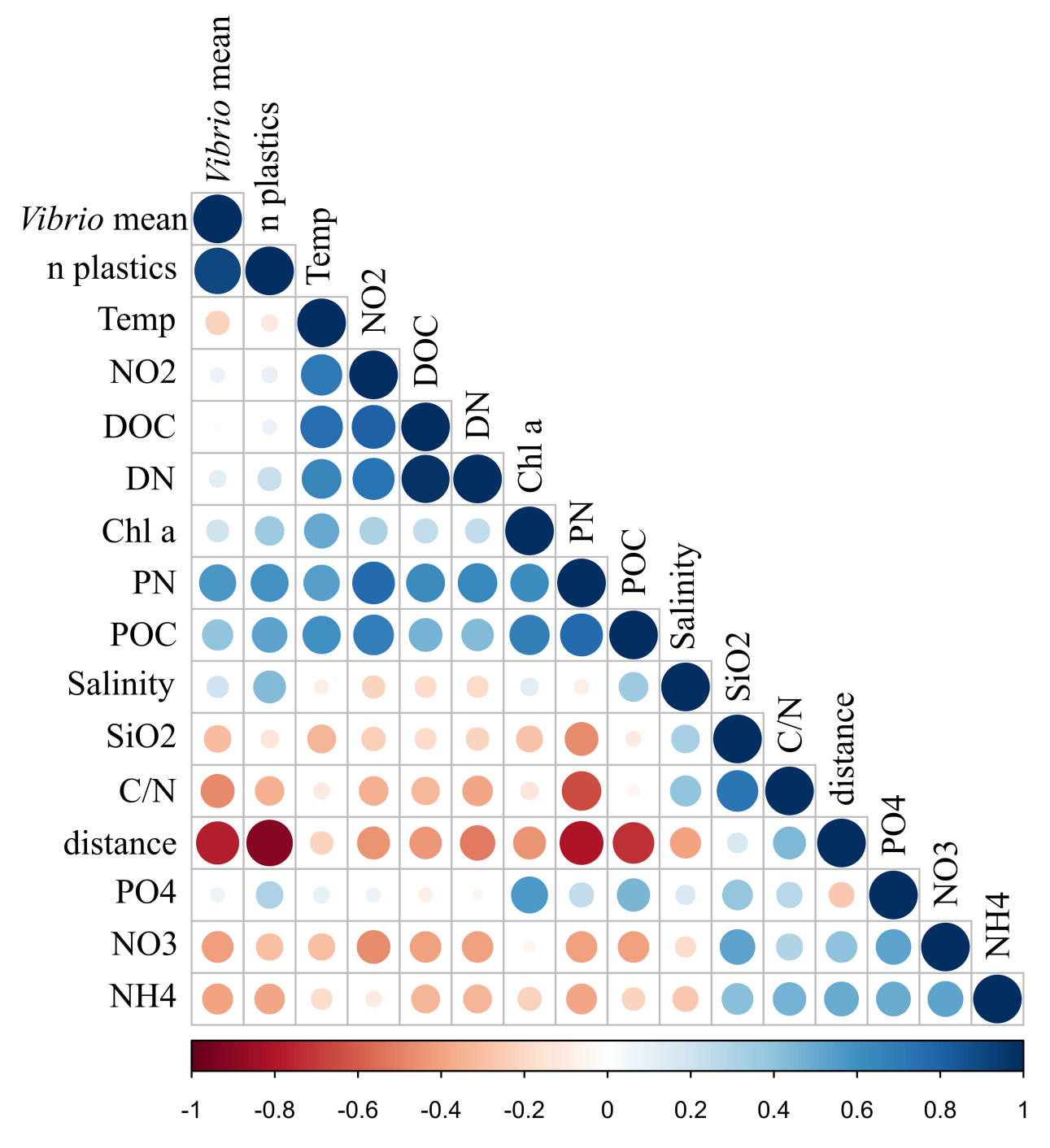


Fig. S4. Spearmann rank correlation (*rho*) of mean *Vibrio* spp. abundances (*Vibiro* mean) per sampling site of field-collected microplastics during a Baltic Sea summer cruise (2015), shortest distance to a major city (distance in km), number of particles (n plastics), physico-chemical water parameters, and nutrient concentrations during sampling. Other abbreviations are as follows: Temp = temperature; NO2 = nitrite; DOC = dissolved organic carbon; DN = dissolved nitrogen; Chl a = Chlorophyll a; = PN = particulate nitrogen; POC = particulate organic carbon; SiO4 = silicate; C/N = carbon-to-nitrogen ratio; PO4 = phosphate; NO3 = nitrate; NH4 = ammonium. The correlogram was constructed using the corrplot-package for R.

Fig. S5a. *Vibrio* relative abundance (%) per OTU and replicate of field-collected microplastics (bottom panel), as well as seston-attached (> 3 µm) and free-living (3-0.22 µm) (top panel) during a Baltic Sea cruise in summer 2015 (POS488). Colors indicate different *Vibrio* OTUs based on 97% sequence similarity. For water communities of station MP25, no data is available. For station location within the Baltic Sea, refer to Fig. S5b.


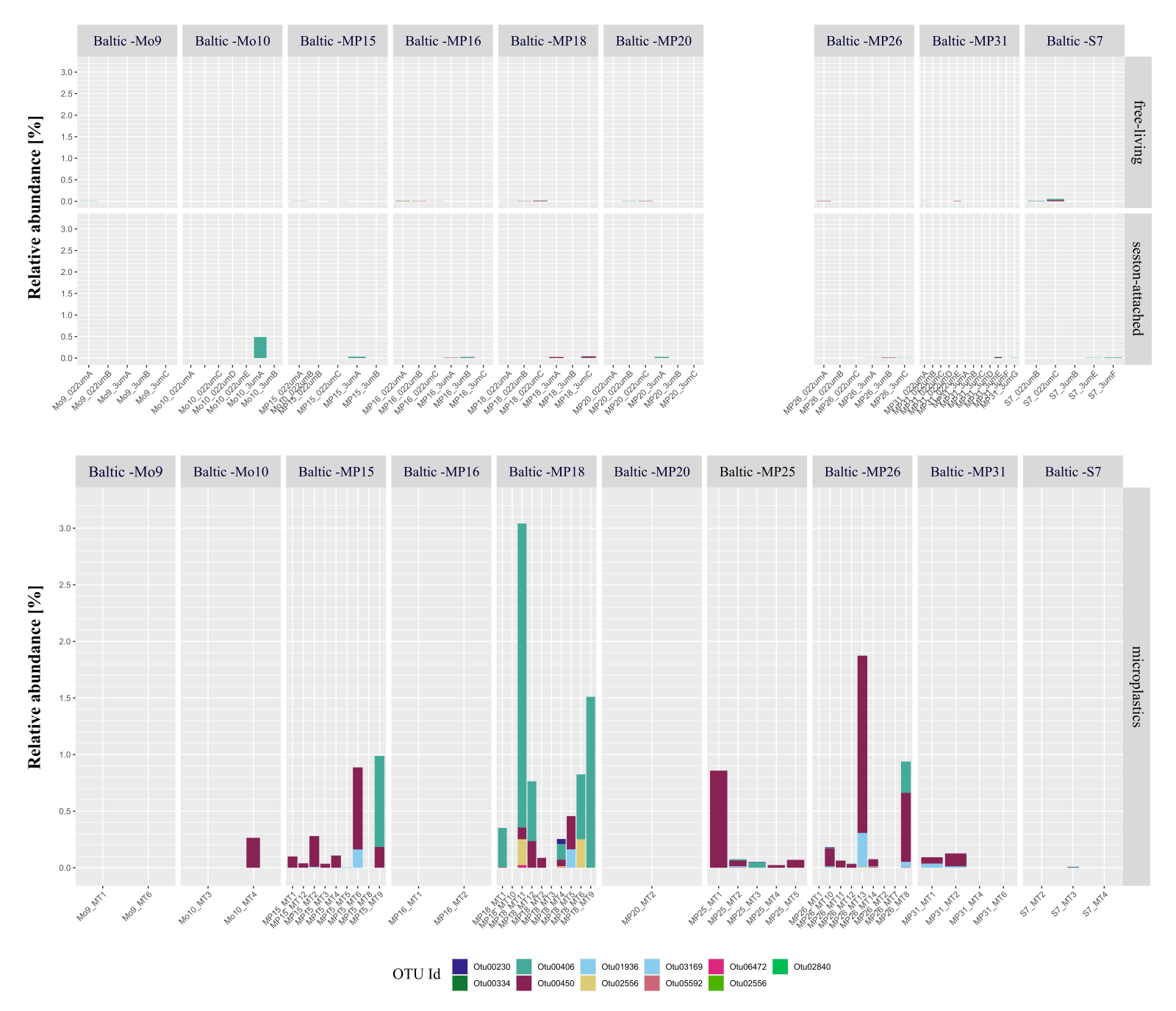


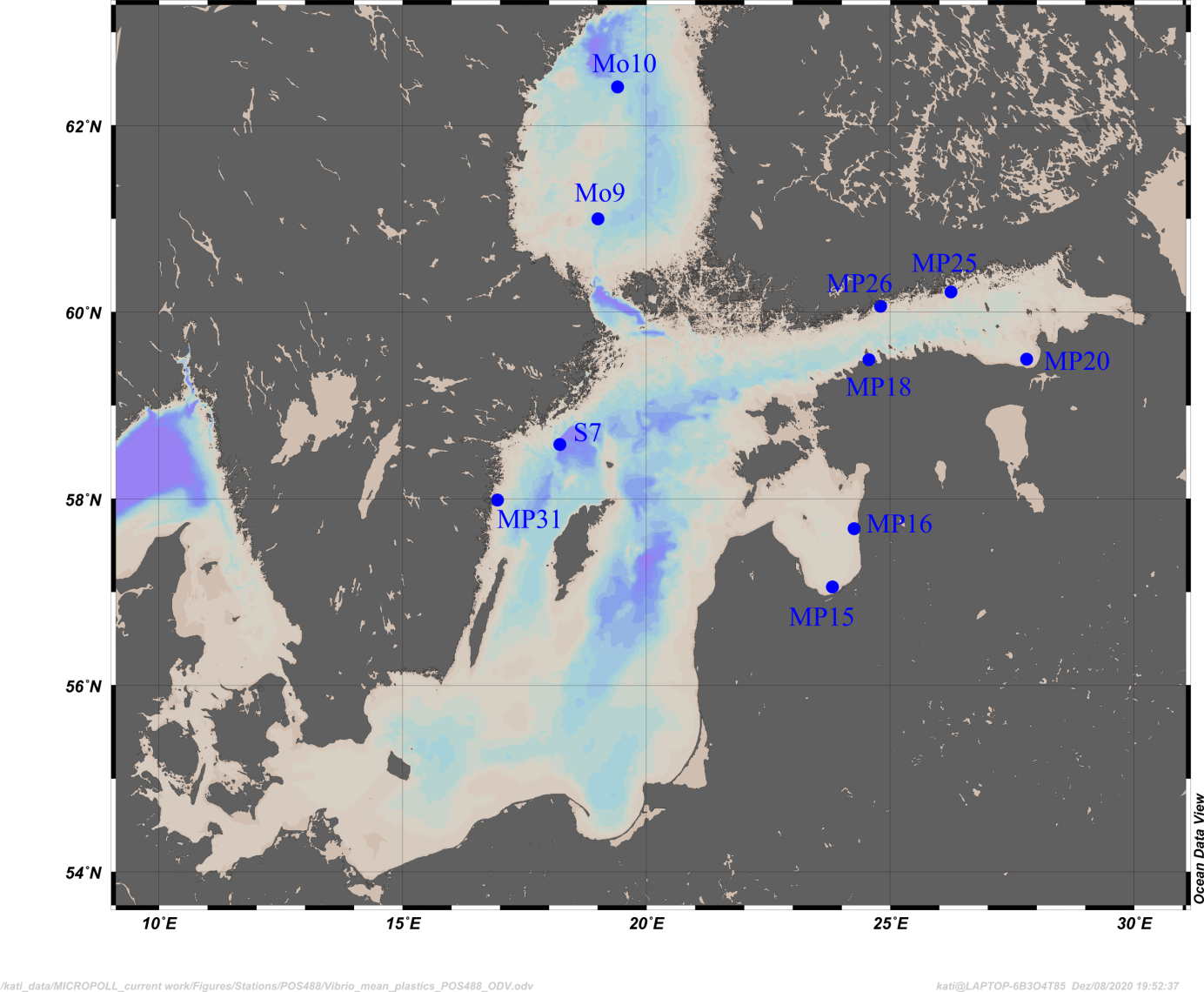


Fig. S5b. Cruise track of the Baltic Sea summer cruise (POS488) with station assignment.

Table S1: Physico-chemical water parameters and nutrient concentrations during the in situ incubation experiment covering the Warnow river (Stadthafen) and 2 Baltic Sea coastal station (Heiligendamm & Hohe Dune). Abbreviations are as follows: Temp = temperature; Sal = salinity; O2 = oxygen; SiO4 = silicate; NO3 = nitrate; NO2 = nitrite; PO4 = phosphate; Chl a = Chlorophyll a; POC = particulate organic carbon; PN = particulate nitrogen; DOC = dissolved organic carbon; DN = dissolved nitrogen. Measures are µmol/L unless otherwise stated.

* these parameters were measured only once.

| Site | Date | Time | Temp °C | Sal  g/kg | O2  % | O2  mL/L | SiO4* | NO3* | NO2* | PO4* | Chla* mg/m^3^ | POC* | PN* | DOC* | DN* |
| --- | --- | --- | --- | --- | --- | --- | --- | --- | --- | --- | --- | --- | --- | --- | --- |
| Stadthafen1 | 09.05.2019 | 09:17 | 12.3 | 6.06 | 78.9 | 7.87 | 25.25 | 8.82 | 0.52 | 0.44 | 4.59 | 129.9 | 16.4 | 470.7 | 47.6 |
| Stadthafen2 | 09.05.2019 | 12:10 | 13.8 | 6.56 | 82.1 | 7.96 | 25.25 | 8.82 | 0.52 | 0.44 | 4.59 | 129.9 | 16.4 | 470.7 | 47.6 |
| Stadthafen3 | 09.05.2019 | 18:00 | 14.2 | 5.76 | 100 | 9.71 | 25.25 | 8.82 | 0.52 | 0.44 | 4.59 | 129.9 | 16.4 | 470.7 | 47.6 |
| Heiligendamm1 | 14.05.2019 | 12:27 | 12.1 | 10.2 | 94.1 | 9.88 | 6.4 | 0.22 | 0.03 | 0.15 | 0.743 | 22 | 2.9 | 295.7 | 16.5 |
| Heiligendamm2 | 14.05.2019 | 13:50 | 12.5 | 10.21 | 95.9 | 9.98 | 6.4 | 0.22 | 0.03 | 0.15 | 0.743 | 22 | 2.9 | 295.7 | 16.5 |
| Heiligendamm3 | 14.05.2019 | 16:50 | 12.7 | 10.56 | 97.2 | 10.07 | 6.4 | 0.22 | 0.03 | 0.15 | 0.743 | 22 | 2.9 | 295.7 | 16.5 |
| Hohe_Dune1 | 17.05.2019 | 09:35 | 12.7 | 10.11 | 89 | 9.01 | 3.95 | 0.49 | 0.07 | 0.42 | 1.238 | 22.3 | 3.1 | 308 | 19.8 |
| Hohe_Dune2 | 17.05.2019 | 12:26 | 12.7 | 10.28 | 88.2 | 8.92 | 3.95 | 0.49 | 0.07 | 0.42 | 1.238 | 22.3 | 3.1 | 308 | 19.8 |
| Hohe_Dune3 | 17.05.2019 | 16:36 | 13.8 | 10.24 | 93.9 | 9.28 | 3.95 | 0.49 | 0.07 | 0.42 | 1.238 | 22.3 | 3.1 | 308 | 19.8 |
